# Overlapping Shoeprint Detection by Edge Detection and Deep Learning

**DOI:** 10.3390/jimaging10080186

**Published:** 2024-07-31

**Authors:** Chengran Li, Ajit Narayanan, Akbar Ghobakhlou

**Affiliations:** School of Engineering, Computer and Mathematical Sciences, Auckland University of Technology, Auckland 1010, New Zealand; akbar.ghobakhlou@aut.ac.nz

**Keywords:** object detection, overlapping shoeprint, edge detection, 2-D image processing

## Abstract

In the field of 2-D image processing and computer vision, accurately detecting and segmenting objects in scenarios where they overlap or are obscured remains a challenge. This difficulty is worse in the analysis of shoeprints used in forensic investigations because they are embedded in noisy environments such as the ground and can be indistinct. Traditional convolutional neural networks (CNNs), despite their success in various image analysis tasks, struggle with accurately delineating overlapping objects due to the complexity of segmenting intertwined textures and boundaries against a background of noise. This study introduces and employs the YOLO (You Only Look Once) model enhanced by edge detection and image segmentation techniques to improve the detection of overlapping shoeprints. By focusing on the critical boundary information between shoeprint textures and the ground, our method demonstrates improvements in sensitivity and precision, achieving confidence levels above 85% for minimally overlapped images and maintaining above 70% for extensively overlapped instances. Heatmaps of convolution layers were generated to show how the network converges towards successful detection using these enhancements. This research may provide a potential methodology for addressing the broader challenge of detecting multiple overlapping objects against noisy backgrounds.

## 1. Introduction

In the realm of 2-D image processing and computer vision, the task of object detection, particularly in scenarios with overlapping or obscured objects, poses a significant challenge. The intricacies and diversities in object shapes, textures, and overlapping patterns contribute to the complexity of accurately identifying and segmenting objects within images. This is in contrast to human perception, where the tendency to three-dimensionality helps to isolate objects in the foreground from objects in the background of the image.

Recent approaches, predominantly relying on convolutional neural networks (CNN) for object detection, have shown a notable degree of success of single objects in various image analysis tasks [1,2,3,4]. However, when faced with overlapping objects in two dimensions, these models’ performances drop, mainly due to the inability to accurately segment the overlapping areas and delineate the underlying object boundaries.

A shoeprint represents the textured image left behind when the sole of a shoe makes contact with a surface in the natural environment [5]. In forensic investigations involving shoeprints left behind at the scene of a crime, a clear and complete texture is important for accurate identification [6,7]. However, in reality, there are frequent instances where two or even multiple textures overlap, leading to a loss of information. Also, real shoeprint images contain non-shoeprint content in the background, such as grit and sand, which can be considered noise. While there has been previous work on images containing single shoeprints in the neural network literature, the task of handling multiple shoeprints against a noisy background in the same image remains a challenge. In an endeavour to achieve target recognition of overlapping shoeprints under intricate noise conditions, we employ contemporary neural network models to evaluate their performance. This study specifically focuses on overlapped shoeprint images, where the task is to separate these shoeprints into separate objects in the presence of noise for the purpose of subsequent identification.

Applications of computer vision to shoeprint images are relatively scarce. The unique, discontinuous texture of shoeprint images and their typical incompleteness pose challenges for the research. The sensitivity of neural networks to images containing single shoeprints has been confirmed in a previous study [8]. In that research, a basic convolutional neural network was utilised, comprising three convolutional layers, three pooling layers, and a softmax layer for classification, totalling a seven-layer structure to determine the presence of shoeprints in images—a fundamental binary classification judgment required prior to identification of which shoe the shoeprint belongs to. Whereas previous research focused on detecting single shoeprints in images, there has been no work on detecting overlapping shoeprints in the same image. The aim of this study is to extend previous work on the detection of single shoeprints to the detection of overlapping shoeprints, where this task becomes more difficult because of the need to ensure that the second shoeprint, which will be under the first and corrupted to some degree by the overlap, is not confused with noise. This is especially the case where the degree of overlap is high, and the second shoeprint is obscured for the most part by the first. The detection of overlapping objects, in general, is a relatively under-explored area of object detection in image analysis, with no currently accepted method for tackling the task of distinguishing two similar-looking and possibly identical objects that are overlapped. For instance, in overlapping shoeprint detection, two different shoes of the same shape but a different type may overlap, or the same shoe may overlap itself a second time. Before the shoeprints can be identified, they need to be separated even if they are identical and also distinguished from non-shoeprints (e.g., pebbles, soil, noise). Approaches based on edge detection and bounding boxes tend to converge to a single box based on confidence thresholds [9]. Such convergence may discard useful information concerning the location of edges of a second object and may fail with highly overlapping objects. Possible applications of successful overlap detection include other areas of forensic study, such as overlapping fingerprints.

The aim of the research below is to evaluate the application of deep learning to overlapping shoeprint detection using bounding boxes with enhanced edge detection methods. The main contributions of this study include (a) a novel dataset containing 200 samples of overlapping shoeprints of various degrees and under different amounts of noise available for use by other researchers working on overlapped image analysis and (b) benchmarks of 85% for detecting partially overlapping shoeprints and 70% for detecting almost fully overlapped samples against which to compare other detection methods for overlapping shoeprints. This dataset and set of benchmarks may also be useful for other researchers to use when working in other areas of overlapped image analysis.

The most critical information for shoeprint detection lies in the boundaries between the shoeprint texture and the ground, which together form a complete shoeprint. Retaining only the edge information of the shoeprints as the sole basis for detection might seem like an extreme method of recognition. However, this approach can more clearly reflect the neural network’s sensitivity when processing edge data. In particular, any detection model must be able to ignore the background noise against which the shoeprint image is taken, especially grit, sand, and mud.

For the research described below, we deployed the YOLO (You Only Look Once) model to detect overlapping shoeprint locations. This model was trained using a dataset containing bounding boxes and subsequently used to delineate the position of shoeprints in new images. Additionally, visualisation techniques were employed, offering insights into neuron activation patterns when processing overlapped images to identify possible reasons for the network’s behaviour.

This paper seeks to elucidate the potential synergies between edge detection and image segmentation in enhancing object detection within overlapping shoeprint images. This research not only aims at advancing the accuracy and efficiency of shoeprint analysis but also extends possible methods for the integration of various image processing techniques in tackling multiple overlapping object detection more broadly.

The Section 2 of this paper provides the study’s background. It discusses the value of shoeprint detection technology, the significance of edge detection techniques, and the various operators used. It also covers the combined application of CNNs and edge detection techniques, as well as the evolution of various YOLO series models. The third part of this paper describes the datasets and methodology, including the sources of the data and how the datasets are generated. It also mentions the evaluation matrix used in this experiment. The Section 4 discusses the experimental process and results, showcasing the performance of the YOLO model under different image pre-processing techniques and analysing the neural sensitivity in different regions using heatmaps. Additionally, it employs a confusion matrix to demonstrate the strengths and weaknesses of the model under two techniques. The Section 5 discusses the labelling, training, and results of this experiment. The final part of this paper concludes this study, discusses the limitations of this work, and outlines future directions.

## 2. Background and Related Work

Shoeprints can provide invaluable clues in the detection of criminal cases [10,11,12,13]. Accurate discriminative features play a critical role in achieving effective performance in shoeprint recognition tasks. The effectiveness of shoeprint detection and identification methods is primarily dependent on the feature extraction technique used, which can exhibit significant variability [14]. A convolutional neural network (CNN) model typically consists of a series of layers that can be trained to recognise patterns in data without the need for prior feature extraction or selection [15].

Edge detection is a pivotal technique in image processing and computer vision, with the objective of identifying the boundaries of objects or regions. Edges typically occur where there is a change in image brightness or colour, signifying the contours of objects [16].

In executing edge detection, techniques such as the Sobel operator, Scharr operator, Prewitt operator, and the Canny edge detection algorithm are often employed to analyse the local structure of the image and determine which points constitute edges [17]. Through edge detection, a binary image can be obtained, in which the white pixels represent the edges in the original image.

Edge detection finds extensive applications in many image-processing tasks such as object recognition [18], tracking [19], segmentation [20], and feature extraction [21], among others [22]. By enabling a better understanding of the structure and content of images, edge detection lays a solid foundation for subsequent image analysis and processing.

There have been many recent advancements in the edge detection technology [23,24]. The application of deep learning and convolutional neural networks (CNNs) has identified new directions for improving edge detection algorithms [25]. Deep learning, with a particular emphasis on convolutional neural networks, has emerged as a new avenue in edge detection research [26]. By training on extensive image data, CNNs are capable of learning edge detection features without prior and separate feature extraction techniques, thereby achieving effective edge detection across a variety of scenarios [27]. The incorporation of attention mechanisms in edge detection models can assist this model in focusing on crucial areas of the image, thereby enhancing the accuracy of edge detection [28]. Through multi-scale feature fusion, edge detection algorithms are able to consider both local and global information of the image, thus enhancing the performance of edge detection [29]. Additionally, researchers have proposed a multitude of optimised network structures to augment the accuracy and efficiency of edge detection, for instance, generating precise edge information through convolutional pyramid features and multi-path aggregation [28]. With increases in computational memory and processing, real-time edge detection has become a reality. This is of significance for applications requiring real-time processing, such as autonomous driving and video surveillance [30].

The evolution of deep neural networks is trending towards increased complexity. To enhance performance in image identification tasks within computer vision, proposed CNN models have become complex, often encompassing millions of parameters [31,32,33]. YOLO (You Only Look Once) stands as a well-known object detection algorithm [34], known for its speed and precision. In contrast to traditional object detection approaches that involve prior feature extraction and selection techniques, YOLO employs a singular neural network model to perform bounding box regression and class label prediction in a single forward pass, achieving its ‘only look once’ effect [35]. Over time, YOLO has undergone numerous iterations and enhancements, leading to the emergence of versions like YOLO 9000 [36], YOLOv3 [37], and YOLOv4 [38]. The study herein utilises the latest in the YOLO series, the YOLOv8 structure, which introduces novel modules, further elevating the model’s usability.

## 3. Data and Methods

There is currently no publicly available dataset of overlapping shoeprints, and so, overlapping shoeprints have to be generated from single shoeprint images from an existing dataset sourced from the German State Criminal Police Offices from Baden-Wuerttemberg, Bayern, Brandenburg, Niedersachsen, and Forensity AG [39]. This dataset comprises 300 original single-shoe images and 1175 single-shoe reference images, with the former being actual photographs of crime scenes depicting shoeprints preserved in soil or on hard surfaces, which were subsequently collected as evidence using gelatine lifters. The reference images, on the other hand, were obtained by scanning the surface of a reference shoe sole covered with gelatine lifters to produce a complete image. Both types of images share a similar generative logic, thus enabling the use of imaging data for model training and testing on the 300 crime scene images. Overlapping images were generated from this dataset, as described below.

### 3.1. Data

The overlapped shoeprint samples are generated by code, with each instance producing distinct features, including noise, shoeprint position, rotation, and overlapping relationships.

Stage 1: Data Generation.

**Figure 1:**
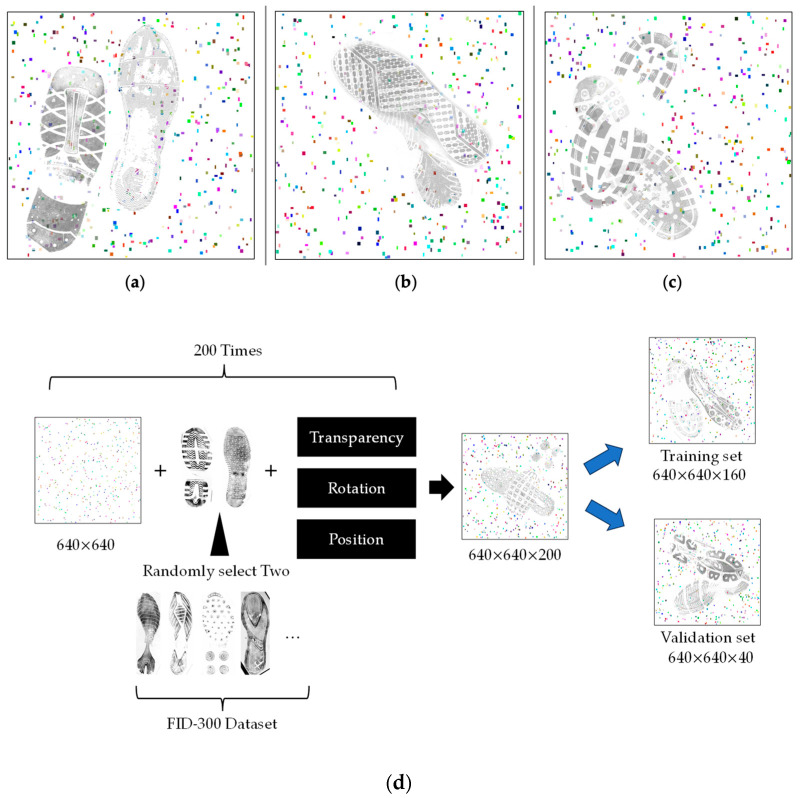
(**a**–**c**) Data generated. Two random shoeprints with 300 to 600 random noise points. (**d**) The pipeline of dataset generated. 1. Create a blank image containing noise. 2. Overlay the image with two randomly selected shoeprint images from the FID-300 dataset. 3. Apply random transparency, rotation, and positioning adjustments to the shoeprints to generate a sample. 4. Repeat the process 200 times, allocating 80% of the samples to the training set and 20% to the validation set.

The primary package used is the Pillow. In the first step, a blank image is created. The image size is 640 × 640, and details regarding shoeprint size, types, and the techniques used to obtain shoeprints have been discussed in previous studies. Please refer to [8]. Approximately 300 to 600 random colour noise points of random sizes ranging from 4 × 6 to 10 × 12 are added to simulate sand and grit (noise). In the second step, random samples are selected from the FID-300 reference folder, and a transparency channel is added with a transparency from 60% to 80%. Through multiple experiments, it was found that images with too low transparency were overly difficult to recognise and did not align with the logic of real samples, while too high transparency would completely obscure underlying pixels, contrary to the conditions of ‘overlapping’ in this task. This step is repeated 1500 times and saved in a new folder named ‘refer_transparent’. In the third step, one photo is randomly selected from the 1800 photos in the FID-300 references folder, and one is randomly chosen from the newly created semi-transparent folder ‘refer_transparent’. The two images are overlapped on the blank base plate containing noise. The position and rotation angle of these two images are kept random. This process is repeated 200 times to obtain an unlabelled dataset (See Figure 1d).

Due to the complete randomness of the samples in this study, the generated images encompass various positional relationships, including those that are not overlapped at all (Figure 1a), those that almost overlap, as shown in Figure 1b, and those partially overlapped, as illustrated in Figure 1c. To validate the sensitivity of neural networks to a wide variety of samples, these non-overlapping samples (Figure 1a) were not removed from this study.

Stage 2: Image Labelling.

**Figure 2:**
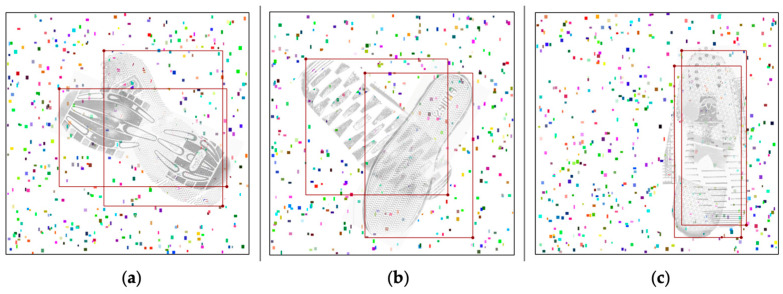
(**a**–**c**) Data after labelling. Two shoeprints are annotated with red rectangle bounding boxes.

The first step in separating shoeprints is the addition of bounding boxes. The image labelling for this study was accomplished using the Labelme annotation software. In this experiment, rectangular bounding boxes were used to annotate the shoeprints. The shoeprints were identified visually, with the annotation boxes extending from the toe to the heel to completely enclose the shoeprints as much as possible. Covered areas were also included in the annotations, aiming to enable the neural network to learn the complex textures of obscured regions. Therefore, there are overlapping areas between two bounding boxes, with some having significantly large overlaps (Figure 2). The annotation files were saved in JSON format and required conversion to TXT documents usable by the YOLO framework through corresponding Python code. In the YOLO format annotation files, the four points of each bounding box are relative positions with the top-left corner of the image as the origin, rather than absolute pixel points.

Sample labelling includes only one category: shoeprints.

The total number of labelled samples for training is 200, divided into two parts: the training set and the validation set, comprising 80% and 20%, respectively, i.e., 160 images for model training and 40 for validation. This test set consists of 20 newly generated images using the method from Stage 1.

### 3.2. Edge Detection

Edge detection is commonly achieved through the computation of image gradients, with the magnitude and direction of the gradients typically used for edge identification. The computation of gradients necessitates the employment of operators. These operators compute the horizontal and vertical gradients of the image through convolution operations, thereby deriving the magnitude and direction of the gradients. The specific formulae are as follows.

The computation of gradients is usually executed by applying operators (such as Sobel, Scharr, or Prewitt operators). These operators, through convolution operations, compute the horizontal and vertical gradients of the image, thus obtaining the magnitude and direction of the gradients. The specific formulae are as follows:

(1) $$\left[G_{x}=I\times K_{x}\right]$$

(2) $$\left[G_{y}=I\times K_{y}\right]$$

where $\left(G_{x}\right)$ and $(G_{y})$ represent the gradients of the image (I) in the *x* and *y* directions respectively; $\left(K_{x}\right)$ and $\left(K_{y}\right)$ denote the convolution kernels in the *x* and *y* directions, and (×) symbolises the convolution operation.

A commonly employed operator for gradient computation is the Sobel operator.

It utilises two 3 × 3 convolution kernels, one estimating the gradient in the horizontal direction and the other in the vertical direction.

The kernel for the horizontal direction is

(3) $$\left[\begin{array}{ccc} -1 & 0 & 1\\ -2 & 0 & 2\\ -1 & 0 & 1 \end{array}\right]$$

The kernel for the vertical direction is

(4) $$\left[\begin{array}{ccc} -1 & -2 & -1\\ 0 & 0 & 0\\ 1 & 2 & 1 \end{array}\right]$$

In routine applications, another operator known as the Scharr operator is also utilised, which holds greater weight in its kernels compared to the Sobel operator, thereby providing more accurate edge detection.

The kernel for the horizontal direction is

(5) $$\left[\begin{array}{ccc} -3 & 0 & 3\\ -10 & 0 & 10\\ -3 & 0 & 3 \end{array}\right]$$

The kernel for the vertical direction is

(6) $$\left[\begin{array}{ccc} -3 & -10 & -3\\ 0 & 0 & 0\\ 3 & 10 & 3 \end{array}\right]$$

The main differences among the Sobel, Scharr, and Prewitt operators lie in the different values of their convolution kernels, which lead to differences in computing image gradients. The variance in kernel values affects the results of edge detection, such as the clarity of edges and the number of edges detected. The Sobel operator is simple to implement and boasts high computational efficiency, capable of detecting edges in both horizontal and vertical directions. However, it is sensitive to noise, prone to false detections in images with a higher level of noise, and may fail to detect diagonal or curved edges [17]. Its edge localisation is not as precise as some other advanced edge detection algorithms [40]. In this experiment, we utilised the Canny algorithm of the Sobel operator [41]. The Canny algorithm initially employs a two-dimensional Gaussian filter to smooth the image for noise reduction, followed by the Sobel operator’s method to compute the image’s gradient magnitude and direction. Hence, the Canny algorithm employs a Gaussian filter in conjunction with a gradient computation method akin to the Sobel operator [41]. The two-dimensional Gaussian function is a common algorithm used for image noise filtering [42]. This function convolves with the image, smoothing each pixel value to alleviate image noise while preserving the image structure. During the convolution process, the new value of each pixel is the weighted average of the values of surrounding pixels, with the weights determined by the Gaussian function:

(7) $$\left[G\left(x,y\right)=\frac{1}{2\pi \sigma^{2}}\exp\left(-\frac{x^{2}+y^{2}}{2\sigma^{2}}\right)\right]$$

where

- (x) and (y) are the coordinates in two-dimensional space;
- $\left(\sigma \right)$ is the standard deviation, controlling the width of the Gaussian function.

Subsequently, Non-Maximum Suppression (NMS) is incorporated for edge thinning, similar to the pooling principle in CNN, selecting only the pixel with the maximum gradient change within a region [43]. After setting maximum and minimum thresholds, gradients exceeding the maximum threshold are deemed as edges, while those below the minimum threshold are considered non-edges. Finally, all strong edge pixels are connected, along with those termed ‘weak edges’, which are adjacent to strong edges and fall between the upper and lower thresholds.

**Figure 3:**
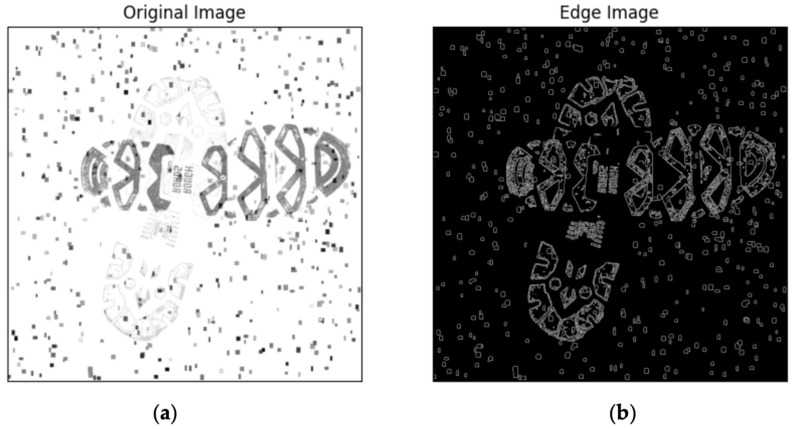
(**a**,**b**) Edge detection image. The Canny algorithm requires only grayscale images to complete edge extraction. Thus, the original colour images are compressed to grayscale images (**a**). Image (**b**) represents the image after edge extraction, where only the edges of the shoeprints and noise surrounding the shoeprint are retained.

**Figure 4:**
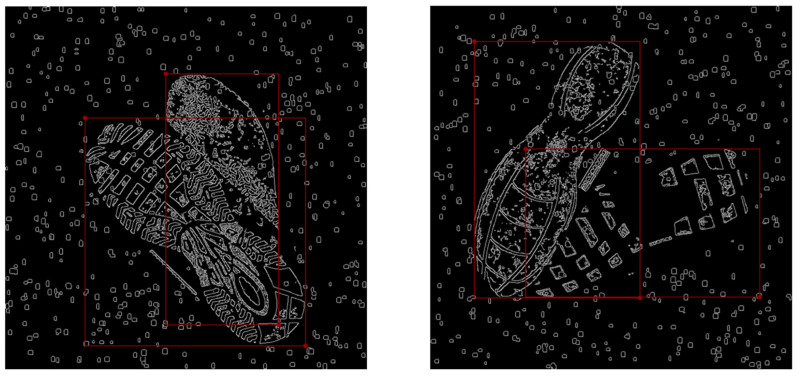
Original labelling with edge images. The annotation red bounding boxes from the original colour images can be directly applied to the edge images and fit appropriately (see above red boxes). These annotated edge images can be directly used for model training.

The image in Figure 3b illustrates a sample of overlapped shoeprints post-application of the Canny algorithm. In comparison to the previous image, all filled pixels have been discarded in this image. After selecting the edges of the image using the Canny algorithm, the newly generated image contains only binary information of ‘edges’ and ‘non-edges’. Unlike the original dataset, which requires three input channels for RGB, the edge image requires only a single channel when inputting into the neural network (here is the YOLO model). This difference needs minor modifications at the input layer of the YOLO model during the experiments. The newly generated image is of identical dimensions to the original image; hence, the annotations from the previous image can be directly applied to the new edge image (see Figure 4).

### 3.3. Object Detection

**Figure 5:**
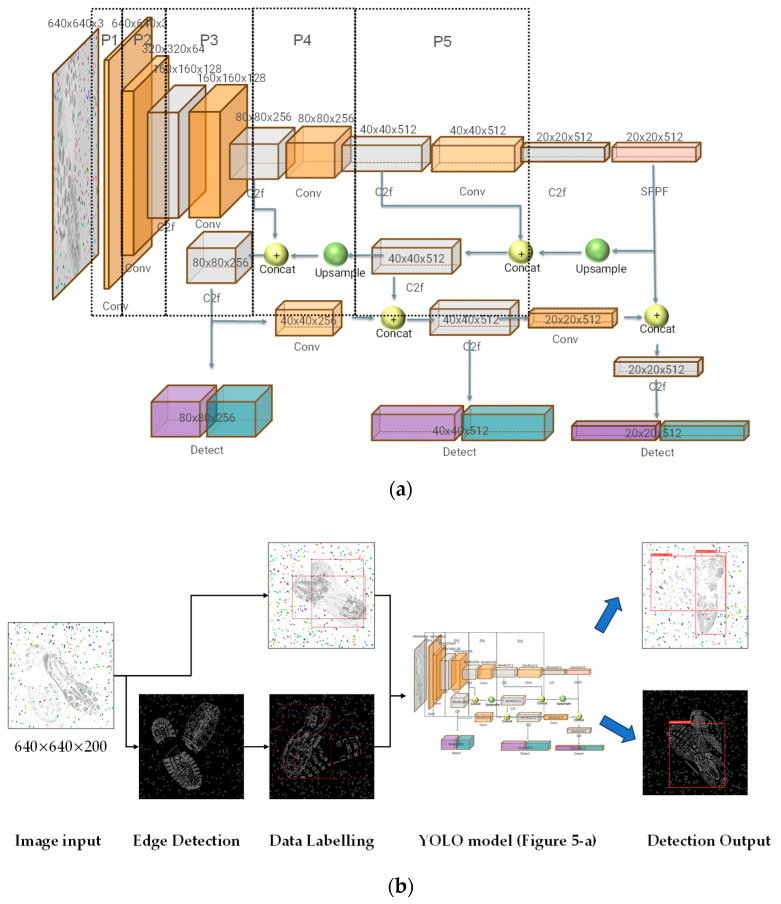
(**a**) The YOLO architecture [44] for overlapping shoeprint detection. Shoeprint samples are fed into the neural network from the left side of (**a**). After traversing to the far right, the network concatenates the feature maps from previous layers (in P4 or P3 stages) with the current layer’s output before proceeding to detect the target. (**b**) Pipeline of this research. 1. The generated dataset of 200 images (640 × 640 pixels) undergoes pre-processing using edge detection technology. 2. Both the edge-detected image dataset and the original dataset are annotated with labels. 3. These datasets serve as input for training convolutional neural networks (CNNs) (details shown in (**a**)). 4. Detection results are subsequently obtained from the trained models (one for original dataset, one for edge detection dataset).

This stage utilises the YOLO v8 model [44]. Due to recent iterations across multiple versions, the neural network’s size and depth have become quite substantial. The model comprises 168 layers with 11,125,971 parameters (see Figure 5a,b). Its primary modules include the Backbone and Head. The model mainly consists of 22 layers (see Table 1), with different layers corresponding to different modules (e.g., Conv, C2f, SPPF). In order to improve the training speed, we selected the v8s version in the v8 model series as the baseline model. Compared with other versions, v8s has the least number of parameters and channels, reducing the total number of model parameters.

To enhance the customisability of large neural networks, this network is designed in modular stages, from P1 to P5, which serve as the main feature extraction phases of the model. Convolutional layers are used as the primary feature extraction layers within these stages. Across different stages, the dimensions and channels of the feature maps vary, but the convolutional kernel size remains fixed at 3 × 3. This choice balances the shortfall in feature collection with larger kernels. Following the convolutional layers, Batch Normalisation operations and the SiLU activation function are applied [45]. Compared to the commonly used ReLU [46] activation function in traditional CNN models, the non-zero centred characteristic of SiLU, its smoother activation curve, and the preservation of information for negative inputs provide a more reliable and comprehensive set of information for the network to proceed to the next training layer.

After the feature extraction through stages P1 to P5, the size of the neural network’s receptive field increases, and each pixel in the feature map represents a larger original size, reaching 32 × 32 pixels. To enhance sensitivity towards detecting and analysing medium and small-sized targets, YOLO incorporates an upsampling mechanism. For identifying medium-sized targets, the small-sized feature map extracted after the P5 stage undergoes upsampling to match the size of the P4 feature map. After concatenating the output of P4, further feature extraction is performed before entering the loss function for target detection. Similarly, a third detection module concatenates the P3 stage to address small targets.

The advantage of this approach is that the output from each feature extraction module in the target detection can be preserved. The fusion of deeper and shallower features can significantly enhance the neural network’s sensitivity when facing complex tasks. A drawback of the traditional AlexCNN [15] is that deeper network models usually have a larger receptive field, which tends to be less sensitive to small targets. Fine edges, textures, and colour features may be lost. However, these details are particularly important for our task.

Among the different layers, the convolutional neural network’s size varies, with dimensions of 20 × 20, 40 × 40, 80 × 80, 160 × 160, and 320 × 320 all being utilised.

C2F is a unique module to YOLOv8, allowing YOLOv8 to obtain richer gradient flow information while ensuring a lightweight structure.

SPPF (Spatial Pyramid Pooling Fast) is a specialised pooling module developed after SPP [47]. SPP replaced the traditional single-layer max-pooling structure, implementing a maximum pooling module without changing the image size. SPPF further optimised the module structure, improving the running speed.

For further details of the full YOLO architecture, including the Backbone and Head, see [40].

### 3.4. Evaluation Matrix

#### 3.4.1. Precision and Recall

For this task, precision (how often the model is correct) and recall (whether all shoeprints are found) are the most important metrics since the task is to identify all possible overlapping shoeprints in images for further identification and classification. Assessment of predictive outcomes is conducted through the quantification of various classification result ratios.

More precisely, precision represents the proportion of samples that are actually correct within all samples predicted as positive. Recall, on the other hand, quantifies the fraction of samples predicted as positive out of all the samples that are inherently positive.

#### 3.4.2. mAP

mAP (mean average precision) is a commonly used metric for evaluating object detection model performance. mAP50 refers to the average precision at an IoU (Intersection over Union) threshold of 0.5. IoU is a metric that measures the degree of overlap between predicted and actual bounding boxes. When IoU is greater than or equal to 0.5, the prediction is considered correct. mAP50–95 is the average precision at different IoU thresholds. Typically, these thresholds range from 0.5 to 0.95, in steps of 0.05. Therefore, mAP50–95 is the average of mAP at these different thresholds, providing a more comprehensive assessment of model performance.

#### 3.4.3. Heatmaps

To investigate the relationship between the image and the activated regions in the neural network, various visualisation techniques were employed. This aimed to observe the sensitivity of certain features (regions) within the neural network. Class Activation Mapping (CAM) is a prevalent image visualisation method that facilitates in-depth analysis of specific layers in deep neural networks [48]. The Class Activation Mapping (CAM) heatmaps illustrate the activation levels across various regions of the network during decision-making processes. The heatmap employs a colour spectrum where red or yellow indicates high activation, and blue signifies low activation. These activation levels suggest the areas of the image that the network deems crucial for object recognition. The activated regions are essential for the successful detection of objects. However, neural networks typically make decisions based on a comprehensive integration of multiple channels. They rely not only on these highly activated areas but also on knowledge learned from other regions based on the overall distribution of features. Moreover, they utilise contextual information, acquiring auxiliary details beyond the important features. Nevertheless, these heatmaps can provide useful insights into how and why neural networks reach convergence in their output at various layers (further details below).

## 4. Experiments and Results

Three experiments were conducted as follows. All experiments were executed on the Google Colab platform, utilising the Nvidia A100 SXM4 40 GB graphics card for training.

(a) In the first experiment (E1), we employed a dataset and set various hyperparameters after initial trial runs to identify effective values. The image size was set at 640 × 640, with a batch size of 16. Dropout was disabled, and the learning rate was set at 0.01, remaining constant throughout the training process. Momentum was set at 0.937, which aids in preventing gradient vanishing and enhances the convergence speed of the algorithm. Weight decay was set to 0.0005 to regularise the model and prevent overfitting. The model underwent training for a total of 1000 epochs. The best results were observed at epoch 360. Training was halted prematurely as no improvement was noted in the last 50 epochs, resulting in 410 training iterations. This is determined by a hyperparameter named ‘patience’, aimed at reducing the duration and cost of training. This hyperparameter’s purpose is to cease training prematurely if no improvements are noted over several epochs. The patience parameter was set at 50. The training duration amounted to 0.2 h;
(b) In the second experiment (E2), we evaluated the difference between the number of training iterations and the final results. We had reservations about the effectiveness of the initial setting of 50 parameters of patience. In this experiment, we eliminated the ‘patience’ hyperparameter, allowing the training to fully reach the initially set 1000 epochs. The total training duration was 0.5 h;
(c) In the third experiment (E3), the aim was to study the performance impact of edge detection technology on the target recognition stage, so the model and hyperparameter settings that performed best in previous experiments, E1 and E2, were selected.

After 410 training iterations, we employed some newly generated samples (test set). The images were roughly of two types: one where two shoeprints were nearly orthogonally overlapped and the other where two shoeprints were almost entirely overlapped. We used the newly trained model to detect the shoeprints from images, and the confidence level thresholds were adjusted to greater than 40%. Below are some samples’ performances in this model.

**Figure 6:**
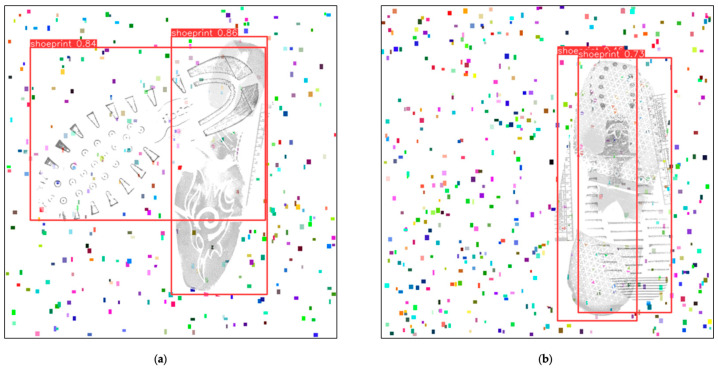
(**a**,**b**) Shoeprint detection using the original dataset. Red bounding boxes indicate the positions of shoeprints detected by YOLO, with the annotation boxes displaying the detected object name ‘shoeprint’ and the confidence of being classified as a true positive. Noise of random colours and positions was used to simulate real-world gravel.

Figure 6a,b reflects two degrees of overlapping images, with minor overlapping and almost complete overlapping. In these two scenarios, the YOLOv8 model achieved an accuracy rate exceeding 85% for samples exemplified in Figure 6a and over 70% for samples exemplified in Figure 6b.

**Figure 7:**
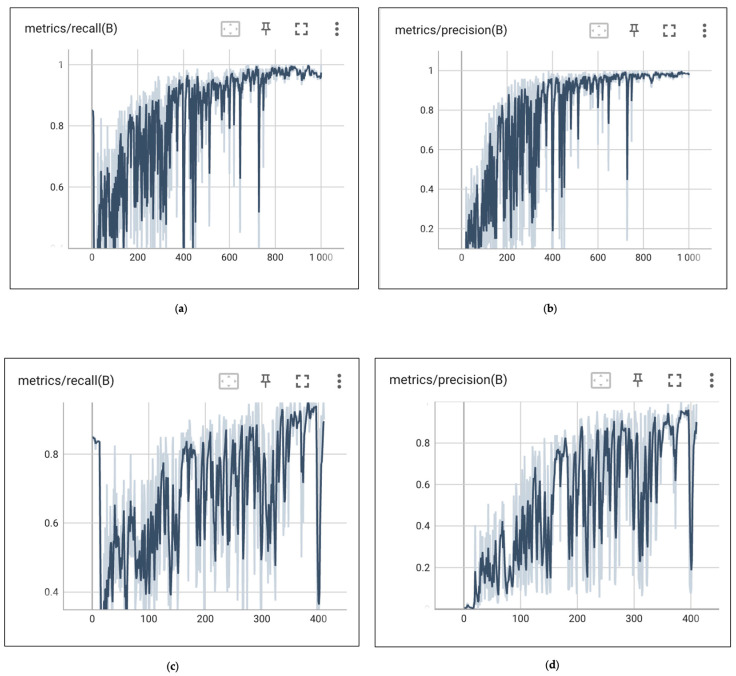
(**a**–**d**) Recall and precision of two experiments strategy. Different training strategies lead to different training curves. Training chat comparation 410–1000 epoch. The *x*-axis of the four charts represents the training epochs, ranging from 0 to 1000 or from 0 to 410. The *y*-axis indicates the values of recall or precision, with a range from 0 to 1. (**a**,**b**) depict the training curves for E2, while (**c**,**d**) illustrate the training curves for E1.

The log curve during the training process displayed some interesting properties. As shown in Figure 7a (Precision) and Figure 7b (Recall), the trend increases with the addition of epochs, but the fluctuations within each epoch are substantial, even oscillating between 0 (worst) and 1 (best). This is due to the varying difficulty brought by different samples, but the specific reasons and training strategies require further study.

In Experiment 2, which underwent a complete training of 1000 iterations, Figure 7a,b shows the fluctuations in precision and recall during the training process. The curves experienced significant fluctuations in the early stages of training, especially before 450 epochs, where the detection precision dropped sharply and then rapidly increased on several occasions. After 500 epochs, the model’s convergence rate decreased, with precision slowly improving.

From Figure 7c,d, which represents 410 training sessions, this model converged very quickly, with over 0.8 in recall and precision around 150 epochs. However, in the context of the current task, the model’s learning rate was not stable, fluctuating between approximately 0.1 and 0.85. However, as evidenced in Table 2, in comparison to Experiment 1, mAP50 has improved slightly from 0.984 to 0.994, and mAP50–95 has increased by 0.055.

**Figure 8:**
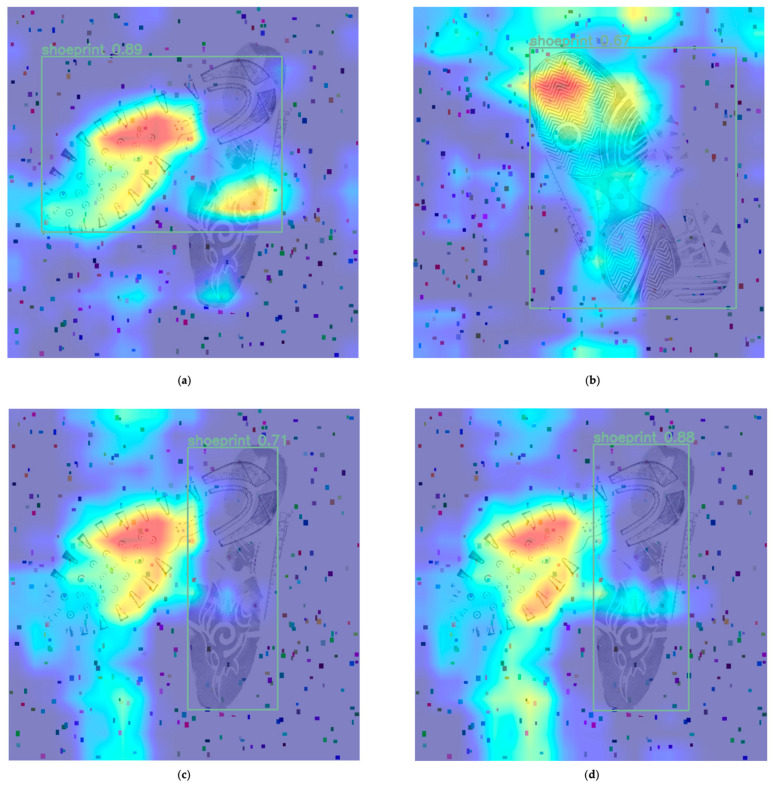
(**a**–**d**) Heatmaps integrated with final detection bounding boxes. Different colours in the heatmap represent the activation level of the region. Red or yellow represents high activation, and blue represents low activation. The heatmaps are extracted from the output of the SPPF module, which is the last module in the backbone part of the YOLOv8 model. After multiple feature extractions, the SPPF module amalgamates features from various scales, representing the comprehensive features extracted from the input image. The positions of the shoeprints are also categorised into two types: the shoeprints in images (**a**,**c**,**d**) have only slight overlaps, while shoeprints in image (**b**) almost completely overlap. The activated areas and the boundary boxes output by the prediction layer nearly superimpose in image (**a**). The shoe print in the upper left shows a relatively concentrated high activation, which may be because this area has unique textures or features required for shoeprint detection. For the bottom shoeprint (right-side shoeprint in images (**c**,**d**)), the correlation between the activated regions and the detection bounding boxes is not evident. Neural networks consider multiple feature maps when making decisions. Even if the activation in some areas is not particularly high, the features of other areas with high activation may be sufficient for the model to detect the entire object.

During the forward propagation process, a neural network generates multiple feature maps, which are the outputs from the last convolutional layer. Each feature map can be viewed as an encoded version of the input image, highlighting specific features within the image. For Class Activation Mapping (CAM), the contribution of each feature map is determined by learned weights. These weights are optimised through the backpropagation algorithm during the training of the neural network, reflecting the importance of each feature map for the final class decision. By multiplying each feature map by its corresponding weight and summing them up, a single ‘activation map’ is obtained. This map displays the most critical regions within the input image. This process is achieved through weighted averaging, ensuring that each feature map contributes appropriately to the outcome based on its importance. Finally, this activation map is usually transformed into a heatmap through colour mapping and superimposed on the original input image. High activation areas may be represented in warm colours (such as red or yellow), while low activation areas in cool colours (such as blue). This colour-region mapping allows us to visually identify which areas are the most crucial for the model when making specific category predictions. YOLOv8 inherently contains multiple hidden layers, yet CAM retrieves a visualisation of one output of the coevolution layer. In this experiment, the heatmaps were generated from the output values of the SPPF module, which is the last module in the backbone of the YOLOv8 model. Having undergone multiple feature extractions, the SPPF module amalgamates features across various scales. The output of the SPPF layer represents a collection of all significant features extracted by the model. The SPPF output size is 40 × 40 × 512; the convolution kernel size is 3 × 3, and the step size is 2. Distinct areas representing shoe shapes can be discerned at this level. It is possible to discern some activated neuron positions, especially around the sole and non-overlapped regions (as seen in Figure 8a). Conversely, when encountering overlapped regions, there is a heightened likelihood that the neurons within that area remain inactivated (as illustrated by the heel area in Figure 8a and the overlapped shoeprints in Figure 8b).

### Result by Edge Detection Method

Table 3 presents the hyperparameter settings for this experiment, utilising the v8s model from the YOLOv8 series to compare the training results of two datasets. This series of hyperparameters was obtained through multiple experiments to ensure that it achieved the best results within the existing model range. Initially, the results were obtained using random hyperparameters, and then, we used grid search optimisation as a hyperparameter optimisation technique to find the best hyperparameter settings for the YOLO model at this stage, shown in Table 3. As discerned from Table 4, employing edge images reduced the epochs required for model convergence from 340 to 230. Although the maximum training epochs were set at 1000, due to the design of the patience value, training would cease upon achieving the optimal results. However, the final test results indicate that the evaluation parameters for the edge image dataset have decreased. The mAP50 dropped from 0.966 to 0.957. For more challenging tests, the mAP50–95 decreased from 0.673 to 0.589. Recall reduced from 0.925 to 0.899. Precision also fell from 0.945 to 0.878.

**Figure 9:**
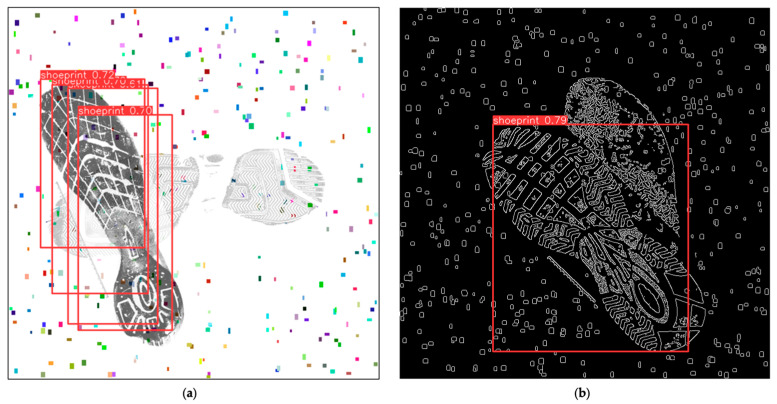
(**a**,**b**) Testing result—shoeprint detection using the edge image dataset. In the detection of the original image (**a**), there were multiple bounding boxes repeatedly detecting the same object, which was a false positive case. Another shoeprint that was partially overlapped was not detected, resulting in a false negative. In the edge-only image (**b**), the red detection box could only detect the position of one shoe, while the other was not detected—another false negative.

The comparison between Figure 6 and Figure 9 shows that problems continue to exist with edge detection in some cases, with only one shoe being recognised out of more than one in the image or multiple recognitions of the same show.

**Figure 10:**
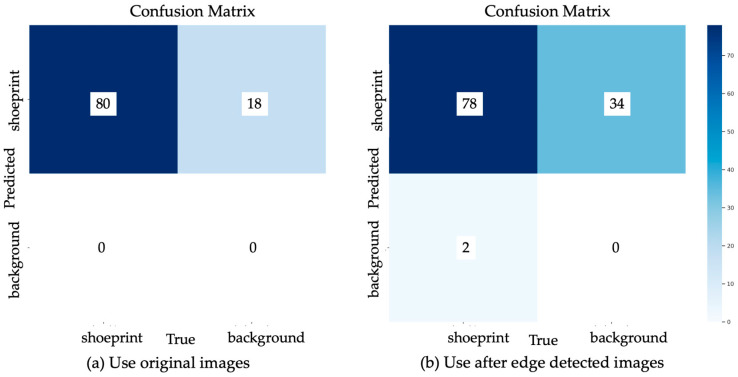
(**a**,**b**) Confusion matrix of two methods. In the validation set, which comprises a total of 40 images containing 80 shoeprints, (**a**) illustrates the performance of the model using the original image dataset under YOLO. It is evident that this model can accurately identify all 80 shoeprints (mAP50) as True Positives. However, it also generates an additional 18 false positives when incomplete areas are mistakenly detected as shoeprints. In comparison, dataset shown in (**b**), which utilises edge detection technology, leads to 34 repeated detections in singular areas (false positives) due to the model’s lower learning performance on complete shoeprints.

The confusion matrices of the two datasets were compared to analyse which stage of the process is responsible for the misclassifications. In Figure 10a,b, it is apparent that the use of edge detection technology (Figure 10b) damages the information in the images, preventing the model from learning effectively. This likely resulted in the 34 false positives observed in Figure 10b, which is higher than the 18 false positives using the original image dataset (Figure 10a). Notably, in Figure 10b, there were two true negatives in the edge-detected image dataset, indicating that two shoeprints were not detected. This may also be due to the loss of colour and texture.

## 5. Discussion

### 5.1. Discussion on Training

As epochs increase, there is an overall enhancement in the model accuracy, with metrics such as mAP, Precision, and Recall steadily rising and losses progressively declining (Figure 7a,c). A sharp decline in accuracy around the 400th epoch is suspected to stem from overfitting. By the time training reaches 1000 epochs, significant fluctuations are still observed around the 700th epoch (Figure 7b). The model’s hyperparameters require optimisation, and the training strategy needs refinement. For this experiment, we set the learning rate at 0.01 and added additional momentum for rapid convergence. Future experiments should further probe the balance between a lower learning rate and cost efficiency to remove the possibility of oscillation. One hyperparameter in this experiment is ‘patience’, aimed at conserving computational resources and avoiding redundant training. Extra training after reaching the evaluation threshold of ‘No improvement’ yields marginal enhancements in model limits yet consumes vast computational resources.

### 5.2. Discussion on Results

The objective of this research was to employ a deep model to pinpoint shoeprint locations. When presented with samples of varying overlapping degrees, the model’s confidence fluctuated, typically ranging from 70% to 85% (Figure 6a,b). The inherent complexity of the images dictated the neural network’s accuracy. Samples that are almost entirely overlapped represent a challenging detection task, and many labelling errors emerge when handling such images. Common issues include marking only one shoeprint or marking beyond two. The heatmap (Figure 8) reveals that the neural network exhibits distinct activation marks on the edges of the shoeprints, yet overlapped regions, owing to their textured covering, remain unlearned. Subsequent predictions then exclusively depend on non-overlapped regions. This is exemplified in Figure 8b, where shoeprints, due to extensive overlapping, are detected as a singular entity by the model, and their bounding box encapsulates both shoeprints. Subsequent training might explore the use of non-overlapped images, essentially raw data. This would ascertain if original samples could achieve comparable or nearly similar outcomes, further reducing research costs.

### 5.3. Discussion on Data Annotation

For this experiment, shoeprints were labelled using rectangular bounding boxes parallel to the image frame. However, shoeprints on photographs present in various orientations lead to a sizable inclusion of noisy regions during image labelling. In this experiment, the proportion of noise was minimal, and the model seemingly remained unaffected by it. Yet, in real-world images, regions beyond the shoeprint edges equate to noise. The impact of extraneous regions on the task remains uncertain. Future research might consider employing rotatable rectangular bounding boxes for shoeprint annotation, effectively minimising the chances of encapsulating noise. Resorting to ‘segmentation’ tasks using polygons is our ultimate research objective.

### 5.4. Discussion on Edge Detection

The results of object recognition tasks using datasets containing only edge images are unfavourable. The Canny algorithm reduces most of the pixel content in the image, leaving only the edge information. In the existing processing steps, the value of the internal information cannot be judged. Deleting these pixels does not bring any benefit to the accuracy of the model, but instead causes a decline in the accuracy rate. As seen in Figure 7, the obscured areas, due to a decline in clarity, saw the textures of overlapped parts significantly fall below the threshold and become discarded. The model struggles to detect the underlying shoeprints based solely on edges.

The computational load decreased, and training speed saw an increase, with training duration dropping from over 15 min to around 9 min. This effectively reduces the complexity of training. However, this set of experiments also suggests that employing edge detection images and the CNN model together does not enhance detection performance; other integrative methods might be required. For instance, overlaying the edges as a layer onto the original image could enhance the clarity of the shoeprint boundaries. Yet, as previously mentioned, the overlapping areas cannot be accurately segmented based on the edge detection algorithm, which adversely impacts our ultimate research objectives.

## 6. Conclusions

To the best of our knowledge, this study is the first to implement a fully supervised neural network model for detecting more than one partially covered shoeprint in images containing overlapping shoeprints and in the presence of noise. Previous research was only capable of detecting the presence of single shoeprints in clear, complete images. However, this study achieves the detection of incomplete and texture-mixed shoeprints in complex environments. The neural network exhibited over 85% confidence for partially obscured samples and over 70% confidence for almost fully covered samples, based on a dataset containing 200 samples. Creating a dataset that can be used by other researchers is also a contribution of this study. This dataset contains a large amount of simulated randomness under noisy conditions, laying the foundation for future applications using real-world photographic data. This dataset will also be made public and can be used for further research on overlapped shoeprints or, even more broadly, in the field of overlapped image analysis. It is anticipated that detection results will improve with the expansion of the database. The heatmap shows the sensitivity of the neural network to different regions. Especially at the boundary between shoe prints and noise, the shoe prints area is activated, and the background area is almost not activated. This verifies the value of edge detection and the possibility of future image segmentation.

Limitation of work. This study serves as the initiation of a new series of research and presents numerous limitations and deficiencies. First, the generated samples did not involve variations in shoeprint scale, and the robustness of the model to changes in shoeprint size remains unexplored. The shoeprint images used in this study were also limited to containing only two shoeprints per image and 200 images in total, and more complex scenarios, as well as larger datasets, will need further exploration. Second, edge detection technology was attempted as an image pre-processing technique for the dataset, and the results degenerated after its application. This study did not consider other image pre-processing techniques or edge detection algorithms, such as equalisation, binarisation, contrast enhancement, image sharpening, denoising, or other edge detection operators, Prewitt, Laplacian, Roberts, etc. Third, the CNN model used was YOLO; this study did not compare the performance differences between various neural network models, nor were specific improvements and modifications made to the YOLO model. Future work could involve comparing multiple models for sensitivity to overlapped images. Fourth, this study only analysed and discussed the performance evaluation matrix of neural networks, without employing more in-depth structural experiments involving ablation analysis. The impact of different neural network modules on handling overlapping textures will be discussed in future studies. Finally, it is not possible to compare and evaluate the results of our method with previous work on overlapping shoeprints since no previous results have been presented, to our knowledge.

This research employed heatmaps as an analytical tool, helping us to understand the sensitivity of shoeprints in different regions. Neurons at covered shoeprint areas were difficult to activate, and accurate detection of these regions could enhance the final classification results. This constitutes an important topic for future research.

Future work includes the following. (1) Research on neural network structures: The YOLOv8 model employed in this experiment is among the most rapidly evolving neural network architectures. Compared to the early stages of the project that utilised a simple seven-layer model [8], consisting only of convolutional and max pooling layers, YOLOv8 has expanded to 168 layers. The necessity and value of more complex structures warrant further exploration. In future research, the relationship between the previous most basic CNN models and contemporary models will be discussed, and the differences between the two baselines will be compared. (2) Changing sample images: The shoeprint images in this study were sourced from the ‘references’ folder of the FID-300 database, where the shoeprints were directly replicated from the manufacturers’ using gelatine lifts, resulting in clear and complete images. The database also contains 300 raw shoeprint samples collected from natural environments such as mud, ceramic, and carpet. The noise from natural sources and the natural incompleteness due to uneven pressure present substantial challenges for the neural network. (3) Once shoeprints are detected, the next stage is to identify (label) the shoeprint against a databank of stored images for forensic investigation purposes. (4) Testing and validating the model on real photographs and images of overlapping shoeprints and comparison of heatmaps for extracted features would form an important validation step of the model in future research.

In summary, employing the YOLO neural network model to detect obscured shoeprints has proven effective, achieving an accuracy of over 70%, with minimal pre-processing of data apart from standard edge detection as required for the bounding box approach. Edge detection as the only pre-processing strategy that allows for the intuitive interpretation of the heatmaps that show progressive extraction of features required for detecting overlapping shoeprints. This lays a foundation for future research, especially in areas where separating objects may be useful in forensic investigations, such as overlapping fingerprints.

## Figures and Tables

**Table 1:** YOLO network workflow from module/layer 0 to 21, including 3 detect modules.

Layers	Type	FeatureMap Name	FeatureMap Size	Channel	Activation Function	Pooling Method
Backbone						
0	Conv	P1	320 × 320	64	SiLU	-
1	Conv	P2	160 × 160	128	SiLU	-
2	C2f	-	160 × 160	128	SiLU	-
3	Conv	P3	80 × 80	256	SiLU	-
4	C2f	-	80 × 80	256	SiLU	-
5	Conv	P4	40 × 40	512	SiLU	-
6	C2f	-	40 × 40	512	SiLU	-
7	Conv	P5	20 × 20	512	SiLU	-
8	C2f		20 × 20	512	SiLU	-
9	SPPF		20 × 20	512	SiLU	MaxPooling
Head						
10	Upsample		40 × 40	512	-	-
11	Concat (From Layer 6)		40 × 40	512	-	-
12	C2f		40 × 40	512	SiLU	-
13	Upsample		80 × 80	512	-	-
14	Concat (From Layer 4)		80 × 80	768	-	-
15	C2f	P3	80 × 80	256	SiLU	-
-	Detect					
16	Conv	P3	40 × 40	256	SiLU	-
17	Concat (From Layer 12)		40 × 40	768	-	-
18	C2f	P4	40 × 40	512	SiLU	-
-	Detect					
19	Conv		20 × 20	512	SiLU	-
20	Concat (From Layer 9)		20 × 20	512	-	-
21	C2f	P5	20 × 20	512	SiLU	
	Detect					

**Table 2:** Two types of training strategy. Comparison of the main result parameters between two different experiments, E1 and E2.

Epochs	Precision	Recall	mAP50	mAP50–95
410	0.989	0.9	0.984	0.71
1000	0.963	1	0.994	0.765

**Table 3:** Hyper-parameter setting in E3.

Model	Epoch Setting	Learning Rate	Momentum	Weight Decay
v8s	1000	0.0001	0.4	0.0005

**Table 4:** Detection result comparison. Comparison of the main result parameters between two different datasets in E3.

Dataset	Epochs Final	mAP_50	mAP_50–95	Recall	Precision
Original Image	340	0.966	0.673	0.925	0.945
Edge Image	230	0.957	0.589	0.899	0.878

## Data Availability

The data that support the findings of this study are available by email from the corresponding author, C.L., upon reasonable request.
